# Late mesenteric ischemia after Sars-Cov-2 infection: case report

**DOI:** 10.1590/1677-5449.200105

**Published:** 2021-03-15

**Authors:** Vilson Sovio Oliveira de Macedo, Geterson Bezerra Moreira, Ana Cristina Fiuza de Albuquerque, Sebastião Carlos de Sousa Oliveira, Mateus Aragão Esmeraldo, Francisco Cesar Barroso Barbosa

**Affiliations:** 1 Universidade Federal do Ceará – UFC, Santa Casa de Misericórdia de Sobral, Sobral, Sobral, CE, Brasil.; 2 Hospital Regional Norte – HRN, Sobral, CE, Brasil.; 3 Universidade Federal do Ceará – UFC, Faculdade de Medicina, Sobral, Sobral, CE, Brasil.

**Keywords:** COVID-19, mesenteric ischemia, laparotomy, anticoagulants, thrombosis, COVID-19, isquemia mesentérica, laparotomia, anticoagulantes, trombose

## Abstract

The purpose of this article is to report the case of a 53-year-old black man, with no previous comorbidities, who presented 48 days after a confirmed diagnosis of COVID-19, complaining of an initially insidious epigastric pain that had progressed to severe pain radiating to the interscapular vertebral region, with hyporexia and episodes of projectile vomiting, with no nausea or fever. Laboratory tests revealed no signs of acute infection or pancreatic injury. Abdominal computed tomography showed dilated, fluid-filled small bowel loops with thickened walls. After clinical treatment, the patient developed persistent abdominal pain. An exploratory laparotomy was performed, finding two sites of small bowel stenosis, with no extrinsic cause, and signs of local ischemia and considerable distension of jejunal and ileal loops. After enterectomy and side-to-side enteroanastomosis, the patient recovered satisfactorily and was discharged with a prescription for oral anticoagulants for outpatient use.

## Resumo

O objetivo deste artigo é relatar o caso de um homem de 53 anos de idade, negro, sem comorbidades prévias, com diagnóstico confirmado de COVID-19 há 48 dias anteriores ao início do quadro de dor epigástrica insidiosa, que evoluiu para dor de forte intensidade que irradiava para região interescapulovertebral, associada a hiporexia e episódios de vômitos em jato, sem náuseas ou febre. Os exames laboratoriais não apresentavam sinais de infecção aguda ou lesão pancreática. A tomografia computadorizada do abdome mostrou alças do intestino delgado dilatadas, cheias de líquido e com paredes espessas. Após terapia de suporte, o paciente evoluiu com dor abdominal persistente. Foi realizada laparotomia exploratória, na qual foram encontrados dois sítios de estenose no intestino delgado sem causa extrínseca, ao lado de sinais de isquemia local e distensão importante das alças jejunais e ileais. Após enterectomia e enteroanastomose primária látero-lateral, o paciente evoluiu de forma satisfatória e recebeu alta hospitalar com prescrição de anticoagulantes orais para uso ambulatorial.

## INTRODUCTION

The outbreak of COVID-19 in the city of Wuhan, China, in December 2019 spread rapidly, reaching the level of a pandemic in early March 2020. A recent report from China identified high inflammatory status as a predictor of unfavorable outcome, suggesting that the higher mortality rate linked to this profile may be due to a state of hyperinflammation triggered by infection by the SARS-CoV-2 virus.^1^^,^ 2

The literature on COVID-19 does not report thrombocytopenia as a clinical feature, but thrombocytosis has been described.^3^ Ranucci et al.^4^ proposed administering antiplatelet agents to patients with critical COVID-19 and high platelet counts, besides increasing the prophylactic dose of low-molecular-weight heparin, noting some clinical benefits.

There is a well-established link between inflammation and increased risk of deep vein thrombosis (DVT). One possible explanation is that inflammation of the vessel wall initiates formation of thrombi, through activation of endothelial cells, platelets and leukocytes that trigger the coagulation pathway.^5^

Moreover, pro-coagulant states have also been recognized for a long time as part of the pathophysiology of acute respiratory distress syndrome (ARDS), as demonstrated by identification of diffuse pulmonary endothelial lesions associated with platelet activation, which could lead to *in situ* formation of macro and micro thrombi that are believed to be embolic. Additionally, the route of interaction between platelet dysfunction, endothelial cells, and neutrophils in ARDS has been associated with development of DVT. Recently, a high prevalence of acute pulmonary embolism has been reported in patients admitted with COVID-19-related pneumonia.^6^

Hospitalized patients with severe infection due to Covid-19 are more prone to excessive activation of clotting, leading to thrombotic events. Tang et al.^7^ discussed the importance of high levels of D-dimer, a degradation product of fibrin, to determination of patients' prognosis and risk of thrombosis. Zhang et al.^8^ described three cases of thrombosis associated with antiphospholipid antibodies, specifically, anticardiolipin antibodies (aCL) and anti-β2-glycoprotein I antibodies (aβ2GPI).

Therefore, the purpose of this article is to report a case of late mesenteric ischemia after infection by SARS-Cov-2. The patient was diagnosed and treated by the Vascular Surgery Service at the Santa Casa de Misericórdia de Sobral, Ceará, Brazil. Ethics approval was issued by the Department of Teaching, Research, and Extension/Continuing Education of the teaching hospital Santa Casa de Misericórdia de Sobral (SCMS) and Ethics Committee in Research of the State University Vale do Acaraú under the registration number 4.404.238. Privacy was guaranteed and the patient was identified by his hospital registration number. Only researchers had access to information on the patient enrolled.

## CASE DESCRIPTION

**Figure 1:**
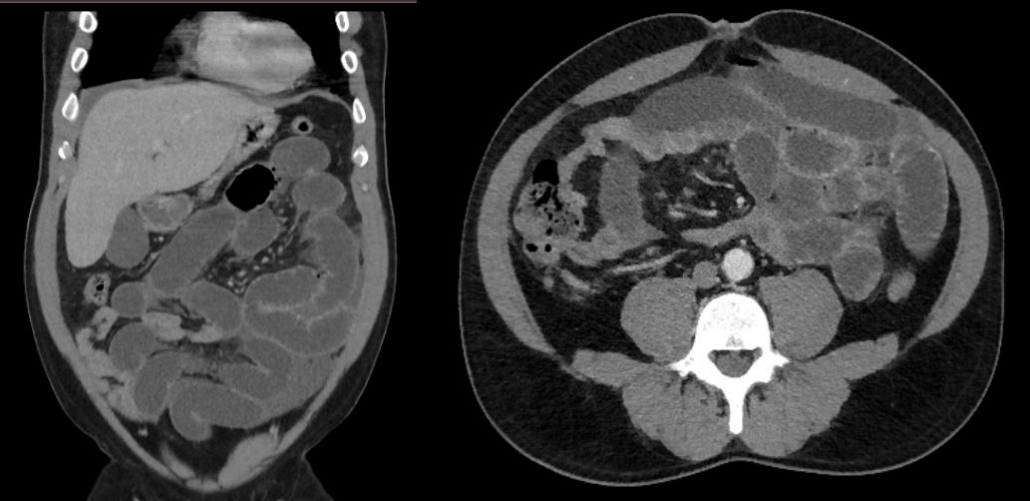
Abdominal Computed Tomography (CT) scan, which showed distended bowel segments and signs suggestive of edema of small bowel loops (thickening of the small bowel wall).

**Figure 2:**
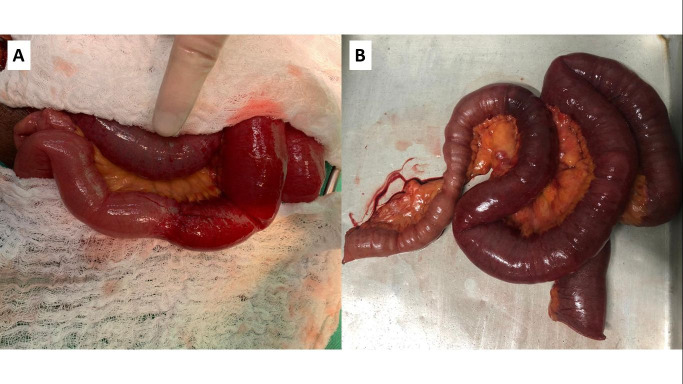
(A) Segment of the small bowel with presence of edema and signs of ischemia, in addition to presence of local stenosis; (B) Resected ischemic intestinal segment.

**Figure 3:**
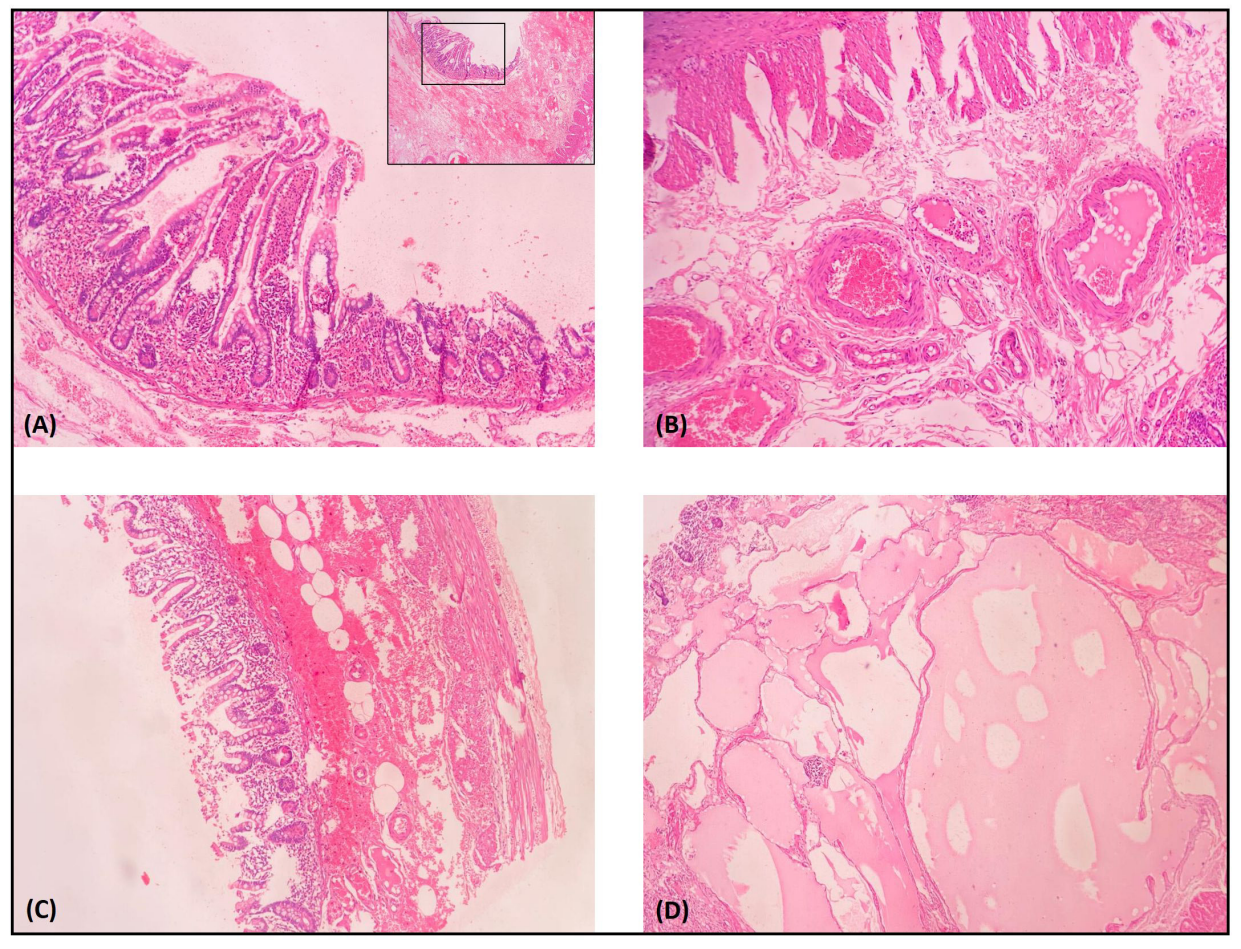
Hematoxylin and eosin-stained sections from the resected small bowel loop. (A) Crypt necrosis with neutrophilic infiltrate; (B) Extensive submucosal hemorrhage (C); Submucosal lymphangioma (D). Arterial and venous submucosal vessels without abnormal histological findings.

A 53-year-old, black, male resident of a small town in the state of Ceará, Brazil, with no history of previous comorbidities, presented with a history of infection by Sars-CoV-2 (positive RT-PCR test) confirmed 48 days previously after contact with an immigrant from the Lombardy region in Italy. During the COVID-19 symptomatic period (fever, dry cough, hyposmia and hypogeusia), the patient remained in isolation and improved from the symptoms. After this period, the patient sought the emergency service, reporting that 1 day before presenting he had started to feel epigastric pain of insidious onset, progressing to severe pain that radiated to the interscapular vertebral region, in addition to hypoxia and 3 episodes of projectile vomiting, but without nausea or fever. At admission, he was hemodynamically stable, eupneic in room air, afebrile, maintaining an antalgic posture, and reporting pain on superficial and deep palpation of the left hypochondrium. Laboratory tests at admission showed no signs of acute infection or signs of pancreatic injury. After admission, the patient underwent abdominal computed tomography, which showed dilated, fluid-filled small bowel loops with thickened walls (Figure 1). After clinical support, the patient developed persistent abdominal pain associated with eructation. A laparotomy was performed and during exploration of the abdominal cavity, considerable distension of jejunal and ileal loops was observed from the angle of Treitz up to 2.3 meters from the ileocecal valve, where it was possible to observe stenosis with no extrinsic compressive cause (Figure 2A). Another stenosis site was observed 80 cm distal of the one previously described, with a clear transition between the proximal ischemic segment and the normal bowel, distal of the second stenosis point. Between the two stenoses, the ileum had an ischemic appearance, with presence of cyanosis. Intestinal pallor was noted, beginning at the angle of Treitz and extending to the first site of bowel stenosis, but still with viable intestinal loops and observable peristalsis. In addition to these findings, there was free fluid in the abdominal cavity. An enterectomy was performed, removing 110 cm of ileum loops with signs of wall thickening, ischemic distress, and two zones of stenosis (Figure 2B). After resection, side-to-side enteroanastomosis was performed with a mechanical surgical stapler. After this procedure, the patient showed signs of clinical improvement. The pathologist’s report confirmed presence of hemorrhagic necrosis of bowel loops, lymphangioma in enteric submucosa, reactive lymphadenopathy, and absence of pathological abnormalities in mesenteric vessels (Figure 3). The results of laboratory tests requested for further investigation of possible causes of intestinal ischemia are shown in Table 1. Additionally, the patient stated that he had no family history of thrombotic events. Transthoracic echocardiogram and computed tomography angiogram of the chest and abdomen were conducted to investigate sources of embolism and vascular occlusion or stenosis. During his postoperative hospital stay, the patient was treated pharmacologically with enoxaparin sodium at a dose of 1 mg/kg administered twice a day, which was subsequently replaced with rivaroxaban 15 mg twice daily for use during outpatient follow-up.

**Table 1:** Laboratory tests to investigate possible coagulopathies in the patient reported with late mesenteric ischemia.

**Laboratory Tests**	**Result**	**Reference Range**
Complement C3 (mg/dl)	183	87-200
Complement C4 (mg/dl)	38	19-52
Ferritin (ng/dL)	416	23.9-336.2
Lupus Anticoagulant	1.18	< 1.2
Cardiolipin IgG and IgM		
IgG (GPL)	9.4	< 15
IgM (MPL)	9.4	< 12.5
Anti-Neutrophil Cytoplasmic Antibodies (ANCAs)		
c-ANCA	Negative	Negative
p-ANCA	Negative	Negative

None of the tests or examinations revealed pathological findings. After recovery from the procedure, the patient progressed satisfactorily and was discharged from the hospital with an oral anticoagulant prescription for outpatient use.

## DISCUSSION

Clinical evaluation must prevail in most situations, including COVID-19. However, laboratory markers may be relevant to raise suspicion of an underlying thrombotic condition in these patients, in addition to good clinical evaluation,^3^ since thrombotic events have been reported in these patients since the emergence of Sars-Cov-2.^6^ Signs of thrombo-hemorrhagic microangiopathy were described in postmortem histopathological analysis reports, with observation of enlarged pulmonary blood vessels containing microthrombi in addition to diffuse thrombotic material in other organs; findings that suggest a multifactorial cause of the thromboembolic events.^9^

However, the peculiarity of the case reported here is that the involvement of intestinal ischemia in this patient happened with no apparent cause. He had no heart disease (arrhythmias, congestive heart failure, or myocardial ischemia), no pathological findings in arteries such as aneurysms or stenoses, and no vascular malformations that could explain the intestinal ischemic event. Regarding the laboratory findings, there was no evidence of autoimmune diseases related to thrombotic events. Despite these findings, antiphospholipid antibody syndrome cannot be completely ruled out in this case, but it is unlikely considering that lupus anticoagulant and IgG and IgM aCL were both negative. While aß2GPI antibody was not measured, which may be a limitation in the description of this case, there is no proven relationship in the literature of the presence this antibody with arterial thrombotic events, which has only been associated with venous thrombotic events.^10^

There are numerous reports that describe associations between COVID-19 and thromboembolic and ischemic events, comorbidities such as obesity and systemic arterial hypertension, and several markers such as antiphospholipid antibodies, factor V Leiden, mutation of the prothrombin or homocysteine gene, etc.^1^^,^ 5 ^-^ 8 Despite this, the case described presented as an infection by COVID-19 followed later by internal ischemia with an interval of 48 days between the two events, with no history of previous comorbidities.

Regarding treatment, although there was no consensus in the literature at the time of submission of this case report, it is believed that there may be benefit to be derived from systemic anticoagulation of patients critically ill due to COVID-19, given the well-established thrombogenic potential of Sars-Cov-2 infection with repercussions for the pulmonary microcirculation.^11^

Laboratory tests for markers of thrombophilia and inflammation were ordered, but the results were within the normal range. The transthoracic echocardiogram and the chest and abdominal computed tomography angiogram that were requested to investigate the possibility of thrombi in cardiac chambers, aneurysms, and arterial stenoses were also normal.

## CONCLUSION

The medical community is still a long way from answering the questions regarding the clinical and laboratory findings that are associated with thrombotic or ischemic events linked to COVID-19. This case report clearly exemplifies this fact, since the patient in the case reported had a recent history of Sars-Cov-2 infection, without comorbidities and with all laboratory and imaging tests negative for suspected thromboembolic and ischemic events, but nevertheless developed late mesenteric ischemia.
